# Bending Test and Numerical Simulation of Externally Prestressed Reinforced Concrete Beams on the Side Facade

**DOI:** 10.3390/ma18133024

**Published:** 2025-06-26

**Authors:** Zhenhua Ren, Ke Zhang, Chengwang Wu, Yi Zhang, Xiantao Zeng, Xuanming Ding

**Affiliations:** 1 Hunan Provincial Key Laboratory of Intelligent Disaster Prevention-Mitigation and Ecological Restoration in Civil Engineering, Hunan Institute of Engineering, Xiangtan 411104, China; zhangke051606@163.com (K.Z.); 17369243690@163.com (C.W.); 13117418934@163.com (Y.Z.); xtzeng63@163.com (X.Z.); 2 College of Civil Engineering, Chongqing University, Chongqing 400045, China; dxmhhu@163.com

**Keywords:** external prestressing, reinforced, concrete beam, bending resistance, ABAQUS

## Abstract

China has a vast number of infrastructure projects, with concrete structures accounting for the majority. To achieve the rapid and effective reinforcement and renovation of existing engineering structures, this paper proposes a novel approach for the rapid strengthening of concrete beams: an external prestressed reinforcement method applied to the side facade. To investigate the effectiveness of this new reinforcement method, we used three ordinary concrete beams serving as control specimens without prestress application, nine beams reinforced using traditional external prestressing, and nine beams reinforced with external prestressing applied to the side facade. The results indicated that, in comparison to the control beam and depending on the initial prestress level, the ultimate bearing capacity of the concrete beams reinforced with traditional external prestressing increased by 152% to 155%. Additionally, for the concrete beams reinforced with external prestressing on the side face, the ultimate bearing capacity improved by 53% to 61%. Both the cracking load and yield load of the reinforced concrete significantly increased, thereby enhancing the overall working performance. Based on the finite element simulation results, it can be observed that the simulation calculation outcomes aligned closely with the experimental test results.

## 1. Introduction

By the end of 2023, China’s civil engineering infrastructure featured a total floor area of existing buildings exceeding 80 billion square meters; approximately 5.4 million kilometers of highways, railways, and high-speed railways combined; and over 1.1 million bridges and more than 38,000 tunnels with a cumulative length of 40,000 km. Notably, the operational mileage of railways reached 159,000 km, while that of high-speed railways was extended to 45,000 km. Bridge engineering constituted approximately 80% of high-speed railway lines. The scale of engineering concrete structures is immense, and the issues of natural deterioration and disaster-induced damage are becoming progressively more severe. The demolition of damaged buildings results in a substantial amount of construction solid waste, leads to resource consumption, and increases carbon emissions. Nevertheless, conventional reinforcement techniques suffer from drawbacks such as stress lag and slow construction progress, and they are incapable of addressing the challenges of emergency repair and reinforcement for structures affected by disasters such as earthquakes and mudslides.

For China, achieving the “dual carbon” goals is not optional but imperative. By applying rapid prestressed reinforcement to existing engineering structures, the functional capacity of these structures can be enhanced and their service life extended. This represents a critical strategy and objective in urban renewal and the optimization of existing infrastructure. Reasonable “life extension” measures are not only capable of stabilizing the “carbon peak” level but also constitute an effective approach toward achieving “carbon neutrality”. In 2024, China decided to implement renovations for an additional one million units in urban villages and dilapidated housing areas.

Currently, reinforcement techniques for engineering concrete structures can be categorized into two types based on whether the original stress state of the components is altered: direct reinforcement and indirect reinforcement [1]. Commonly employed methods for reinforcing reinforced concrete beams include section enlargement, steel bonding, steel cladding, and carbon fiber reinforcement. The external prestressed reinforcement method is a technique that employs high-performance materials as the force-transmitting medium to apply external prestress to engineering structures. This prestress partially offsets the internal forces induced by external loads, thereby enhancing the service performance of engineering structures and increasing their load-bearing capacity. This reinforcement approach has threefold effects: strengthening the structure, unloading it, and redistributing its internal forces [2,3].

In recent years, driven by the widespread application of high-performance materials, a range of external prestressed reinforcement technologies for strengthening engineering concrete structures have been developed. These encompass the in-depth advancement of traditional prestressed reinforcement techniques, the innovation of novel external prestressing methods, and investigations into advanced prestressed materials and structures. The underlying developmental logic involves reinforcing the tensile zones of concrete beams through the external attachment of high-strength material plates, the external anchoring of high-performance fiber fabrics, and the embedding of high-performance reinforcement materials within the tensile zone of concrete for enhanced performance.

Wang Yongbao et al. [4] investigated the static characteristics of concrete beams following external prestressing reinforcement. Qiang Xuhong et al. [5] reinforced a 24 m span railway bridge using in vitro prestressed Carbon Fiber Reinforced Polymer (CFRP) reinforcement and performed numerical simulations and parameter analyses using ABAQUS (2023) software. Li Tang [6] investigated a novel externally prestressed reinforced structure, performed an experimental study on the flexural behavior of damaged concrete T-beams after being reinforced with an externally prestressed truss combination, proposed a new bridge reinforcement technique—externally prestressed truss combined reinforcement—and analyzed the flexural performance of damaged concrete T-beams following the application of this reinforcement method.

Cao Mingyang [7] conducted a numerical simulation and performance prediction regarding the static load and fatigue behavior of reinforced concrete (RC) beams strengthened with prestressed carbon-fiber-reinforced polymer (CFRP) slabs. Using RC beams strengthened with prestressed CFRP slabs as the research subject, a finite element model was established. The study investigated the effects of various factors, including the prestress level and the load-holding level prior to reinforcement, on the flexural performance of the strengthened beams. Zhou Chang et al. [8] conducted experimental studies on prestressed CFRP sheet shear-reinforced reinforced concrete T-beams and performed tests on prestressed CFRP sheet shear-reinforced reinforced concrete T-section beams, thereby validating the effectiveness of the shear capacity model for the strengthened beams. Shu Shen yunhao [9] investigated the mechanical behavior of reinforced concrete (RC) beams strengthened with prestressed carbon-fiber-reinforced polymer (CFRP) under natural exposure conditions. The study aimed to evaluate the reinforcement efficacy of prestressed CFRP materials for RC bridges in a subtropical natural exposure environment. Liu Weihua [10] conducted a study on the mechanical properties of concrete beams reinforced with prestressed CFRP plates, utilizing U-shaped band end anchors and mid-span tensioning strings. The research proposed an innovative technology for reinforcing reinforced concrete beams through the integration of prestressed carbon plates, U-shaped band end anchors, and mid-span tensioning strings. Wang Haitao [11] and colleagues investigated the influence of anchoring methods and prestress levels on the flexural behavior of reinforced concrete beams strengthened with CFRP plates. The findings revealed that the anchoring method significantly affected the ultimate load capacity of the RC beams, while exerting minimal influence on the cracking loads and yield loads. Zhang Zhimei et al. [12] performed a finite element analysis to investigate the flexural fatigue resistance of reinforced concrete beams strengthened with externally bonded prestressed CFRP sheets. Ren Wei et al. [13] explored a calculation method for determining the optimal prestress in prestressed TRM (fiber fabric mesh-reinforced cement polymer mortar) reinforced concrete beams, thereby broadening the application range of TRM technology in the reinforcement of such beams.

Li Yan et al. [14] performed a finite element analysis of the flexural behavior of reinforced concrete (RC) beams strengthened with prestressed high-strength steel strands. Using the ABAQUS simulation software, they modeled four groups of simply supported RC beams under varying operational conditions. These beams were externally prestressed, and their load application processes were subsequently simulated. Shaise K. John et al. [15] conducted seismic reinforcement of reinforced concrete (RC) columns using a fiber-reinforced cementitious matrix (FRCM)/mortar and evaluated its effectiveness by integrating the results of cyclic load tests with numerical analysis methods. Their studies demonstrated that the ductility, stiffness, and ultimate strength of the retrofitted RC columns were significantly enhanced, suggesting that the FRCM system can serve as an effective solution for seismic retrofitting. Americo Cunha et al. [16] investigated the failure theories of Tresca and von Mises, focusing on their adaptability within a probabilistic framework. The study revealed that, from a deterministic perspective, the Tresca criterion can be considered more conservative than the von Mises criterion. However, in a probabilistic context, the von Mises criterion produces lower equivalent stress values compared to Tresca, thereby demonstrating greater conservatism.

Tao Jia [17] investigated the shear behavior of reinforced concrete (RC) beams strengthened with prestressed fiber-reinforced polymer (FRP) grids. To address the degraded shear performance of RC beams, a FRP grid-polymer mortar reinforcement technique was developed. A systematic analysis was conducted on the factors influencing the shear strengthening effectiveness of FRP grids. Additionally, an attempt was made to apply prestress as a method to mitigate the issues of low material utilization and delayed load transfer in FRP grids. Dong Zhuo [18] investigated the flexural behavior of reinforced concrete beams strengthened with prestressed BFRP grids. Considering the high strength and low elastic modulus characteristics of FRP, the novel prestressed tensioning theory applied in the experiment proved to be highly effective. By utilizing strain to inversely control stress, the method successfully mitigated the impact of friction on prestress during the tensioning process. Additionally, it minimized prestress losses caused by anchorage deformation and concrete elastic shrinkage.

Xiao Yunxin [19] conducted a static performance test on end-embedded prestressed CFRP-concrete beams. In response to the challenges of poor bonding performance at the interface between FRP and concrete in externally mounted systems, the need for metal anchors at the FRP ends, and the tendency for concrete protective layers to peel off or fail at the plate ends in surface-mounted systems—issues that necessitate large-scale grooves and may cause secondary structural damage—a novel end-embedding technology was proposed. Peng Hui et al. [20] performed experiments on the shear strengthening of reinforced concrete T-beams using prestressed near-surface-mounted (NSM) CFRP. They introduced a method for reinforcing the web of reinforced concrete beams with prestressed NSM CFRP, developed specialized fixtures and tensioning anchoring devices, and successfully achieved shear strengthening of the web of reinforced concrete T-beams using prestressed NSM CFRP.

Jingjie Wei et al. [21] conducted a study on the flexural performance of Reinforced Concrete (RC) beams strengthened with Textile Reinforced Concrete (TRC) composites embedded with prestressed Carbon-Fiber-Reinforced Polymer (CFRP). The findings indicated that the ultimate load capacity of the strengthened beams increased slightly with an increase in groove size. Furthermore, the increase became more significant as the diameter of the CFRP bars increased. Peng Hui [22] proposed a novel method for reinforcing concrete beams using prestressed FRP near-end-enhanced embedment (NEEE). This approach aimed to address the challenges associated with the engineering application of FRP reinforcement technology, such as high anchorage costs, complex grooving procedures, and the tendency for the protective layer at the ends of CFRP plates to peel off.

Yuxuan Wang et al. [23] conducted an in-depth analysis and calculation of the crack behavior of prestressed high-performance aluminum alloy bars embedded in reinforced concrete beams. Their research demonstrated that the use of aluminum alloy bars as embedded reinforcement significantly enhances the load-bearing capacity of concrete beams. Furthermore, applying prestress, increasing the amount of reinforcement, and elevating the prestress level are all effective strategies for controlling crack propagation, thereby reducing crack width and spacing.

In addition to reinforcing prestressed slabs and prestressed tendons, researchers have also developed a method where “existing beams” and “newly constructed beams” function collaboratively. For large-span concrete beams, the prestressed “new beams” are laterally overlaid onto the “existing beams”, allowing the new and existing beams to effectively share the load following reinforcement.

R. Kirthiga et al. [24] systematically analyzed the technology of prestressed lateral superimposition reinforcement for concrete beams and its practical engineering applications. Based on a case study of a concrete frame structure project in Hangzhou, they proposed an innovative method involving prestressed lateral superimposition reinforcement for concrete beams. Xiang Aijun [25] conducted an in-depth investigation into the flexural performance of long-span concrete beams reinforced using post-prestressed side superimposed beams. Both the prestressed reinforcement technique and the application of reinforced concrete composite beams were demonstrated to significantly enhance the structural performance of long-span reinforced concrete beams.

The reinforcement of concrete columns using prefabricated prestressed semi-circular steel plates has been investigated. Ren Zhenhua et al. [26,27] conducted a study on the compressive bearing capacity of concrete columns reinforced with prestressed semi-circular steel plates. Their findings indicate that the application of prestress to a steel casing composed of two semi-circular steel plates can enhance the axial compressive bearing capacity of concrete columns by over 40%.

Reinforcing existing engineering structures can enhance their reliability, prolong their service life, broaden their application scope, preserve the cultural significance of the structures, and optimize the allocation of social resources.

After conducting a comprehensive analysis, summarization, and generalization of the technical advantages and existing limitations of traditional external prestressing methods, the authors developed an innovative approach involving side-facade external prestressing to enhance the load-bearing capacity of reinforced concrete beams (Patent No. ZL 2019 2 1264085.9). This method effectively addresses the issue of structural integrity that arises from drilling holes in the dense stirrup regions when using conventional techniques. It improves the tensioning efficiency of prestressed steel materials, while reducing the required amplitude of prestress application. Furthermore, by directly securing the tensioned steel bars with bolts, the installation process is significantly simplified, thereby enhancing construction convenience and practicality. In this study, a bending test was conducted on a reinforced concrete beam after external prestressing reinforcement on the side facade, to investigate its failure mode and flexural performance. Using ABAQUS software, finite element analysis of the tested beam was performed to examine its failure mode, load-bearing capacity, and stress and strain distribution. The results were compared with experimental data to provide theoretical guidance for the engineering application of this technology.

## 2. Experimental Program

### 2.1. Materials and Its Properties

**Concrete.** The test utilized ordinary Portland cement concrete with a design strength grade of C30 (Xiangtan, China). The compressive strength of the standard test block under standard curing conditions after 28 days was measured at 35.97 MPa. The compressive strength test for concrete was conducted in accordance with the (GB/T50081-2019) [28].

**Reinforcing bars.** Reinforcing bars were utilized in the construction, including both longitudinal bars and stirrups made of HRB400 steel (Jiujiang Jiangxi, China). Specifically, the lower longitudinal bar had a diameter of 14 mm, with a yield strength of 443 MPa, ultimate strength of 638 MPa, and an elastic modulus of 202 GPa. The upper longitudinal bar (supporting bar) had a diameter of 10 mm, featuring a yield strength of 451 MPa, ultimate strength of 626 MPa, and an identical elastic modulus of 202 GPa. The stirrup, with a diameter of 8 mm, exhibited a yield strength of 445 MPa, ultimate strength of 622 MPa, and an elastic modulus of 201 GPa. The tensile tests for these reinforcing bars were conducted in accordance with the standard (GB/T50010-2010) [29].

**Prestressed tendons.** Prestressed tendons can be categorized into two types. In the traditional prestressed reinforcement scheme, the prestressed tendons consisted of HRB400 (Jiujiang, Jiangxi, China) steel bars with a diameter of 14 mm. These bars exhibited a yield strength of 440 MPa, an ultimate tensile strength of 635 MPa, and an elastic modulus of 201 GPa. In contrast, the external prestressed reinforcement scheme for the side facade employed 14 mm long bolts graded at 8.8, which possessed a yield strength of 659 MPa, an ultimate tensile strength of 783 MPa, and an elastic modulus of 201 GPa.

### 2.2. Component Design and Manufacture

**Figure 1:**
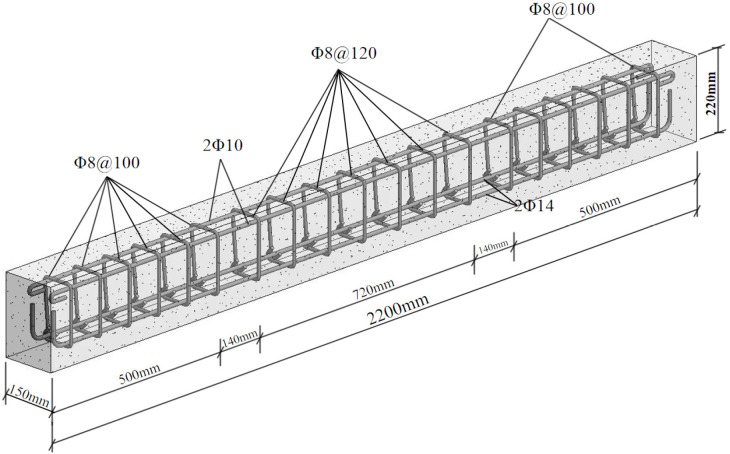
Reinforcement detailing.

**Figure 2:**
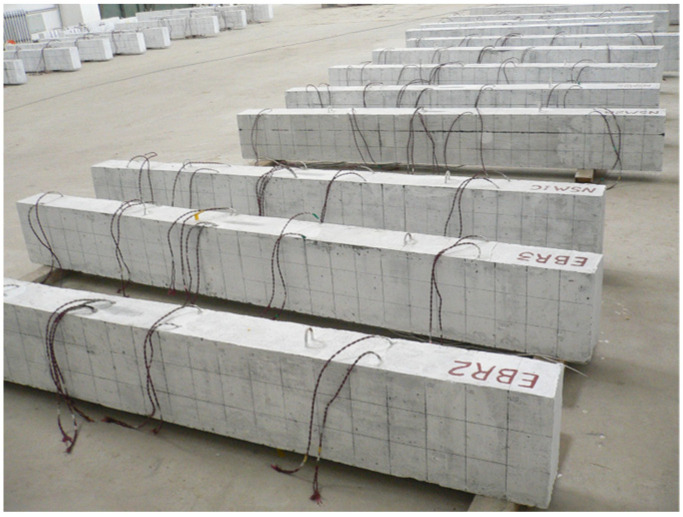
The cast concrete beam.

A total of 21 reinforced concrete beams were designed in this experiment, including 3 Ordinary Beams (OB), 9 Traditional Prestressed Reinforced Concrete Beams (TRB), and 9 Side-Elevation Externally Prestressed Reinforced Concrete Beams (SRB). All test beams featured rectangular cross-sections with external dimensions of 150 mm × 220 mm × 2200 mm. The clear protective layer thickness for the lower longitudinal bars was ***c*** = 22 mm, $a_{s}$ = 37 mm, and the clear protective layer thickness of the upper longitudinal reinforcement was $c^{\prime }=17\mathrm{mm},\;{a^{\prime }}_{s}=29\mathrm{mm}$, resulting in an effective height *h*_0_ of 183 mm for the concrete beam. *C*8@120 stirrups were utilized in the middle third of the beam span, whereas *C*8@100 stirrups were employed at the ends of the beam near the supports within the one-third span region. The reinforcement details are illustrated in Figure 1, and the completed casting of the test beams is shown in Figure 2.

### 2.3. The Prestressing Application Scheme

There are two prestressed reinforcement schemes under consideration. The first scheme involved a traditional externally prestressed reinforced beam, which was subjected to different initial prestress values of 30 kN, 35 kN, and 40 kN. The second scheme pertained to an externally prestressed reinforced beam applied on the side facade, with initial prestress values of 50 kN, 75 kN, and 100 kN. Each reinforcement scheme comprised nine reinforced beams. Both schemes were further divided into three groups, each containing three specimens for comparative testing. The effective prestress and ultimate stress were automatically recorded using a static signal acquisition and analysis system.

**Figure 3:**
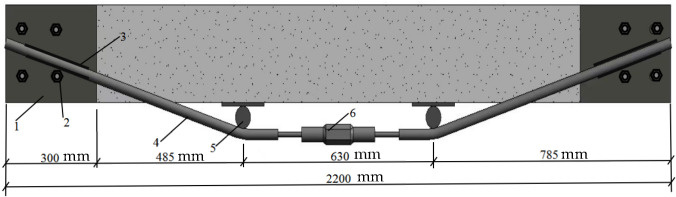
Schematic diagram of the conventional external prestressed reinforcement method. 1—anchored steel plate; 2—high-strength steel plate anchor bolts; 3—foot weld seam; 4—prestressed steel bars; 5—support stick; 6—bidirectional fastening device.

**Figure 4:**
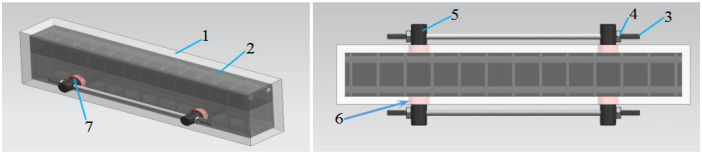
Schematic diagram of the external prestressed reinforcement system. 1—concrete beam; 2—steel reinforcement cage; 3—prestressed steel bars; 4—nut; 5—steel bar; 6—casing hole; 7—steel casing.

As depicted in Figure 3, this represents the conventional externally prestressed reinforcement scheme. Prior to concrete pouring, steel sleeves were welded and securely fixed to the steel cages located at both ends of the beam. Once the beam’s concrete had solidified, four steel plates were affixed to the lateral face of the beam via bolts passing through the steel sleeves at each end. Subsequently, the prestressed steel bars were anchored to plate 1 through welding 3, and support rods 5 were installed between the prestressed steel bars and the underside of the beam. The prestressed steel bars were then tensioned using a bidirectional fastener 6, thereby enabling the application of external prestress. Depicted in Figure 4 is the external prestressed reinforcement scheme applied to the side facade. In the tension zone of the beam, casing hole 6 was created symmetrically between the two vertical stirrups at the midline of the beam span. Inner steel sleeves (labeled as item 7) were tightly inserted into these holes, and steel bars (item 5) were subsequently installed within the sleeves. Following the installation of the steel bars, tensioned reinforcing bars with threaded ends (item 3) were inserted into the circular openings of the steel bars. These tensioned reinforcing bars were then tightened using a torque wrench and secured with nuts (item 4). As a result, the concrete beam on the side facade was subjected to external prestress, which acted in a direction opposite to the primary tensile stress of the beam itself. This effectively enhanced the beam’s working performance and increased its load-bearing capacity.

### 2.4. Measurement Point Arrangement and Loading Method

The test measurements and the layout of measurement points primarily encompassed the following aspects [23,24]:

① A 50 t pressure sensor was installed beneath the loading jack and connected to a static signal acquisition instrument. This setup enabled the computer to automatically measure the load applied to the test beam.

② Dial indicators were positioned at the supports on both ends and at the mid-span location of the test beam for deflection measurement, with real-time recording of the dial indicator data.

③ Three BE120-3AA strain gauges were affixed to each of the upper and lower longitudinal bars within the beam. Additionally, one strain gauge was attached to the force-bearing reinforcement at each three-quarter point of the pure bending section.

④ A BE120-3AA strain gauge was attached at the midpoint of each prestressed tendon and connected to the DH3818Y static signal acquisition instrument (Nanjing, China) to regulate the application of prestress and collect the strain data of the prestressed tendons during the loading process.

⑤ On both sides of the mid-span of the test beam, three BZ120-80AA strain gauges were evenly distributed along the horizontal height to measure the strain of the concrete.

**Figure 5:**
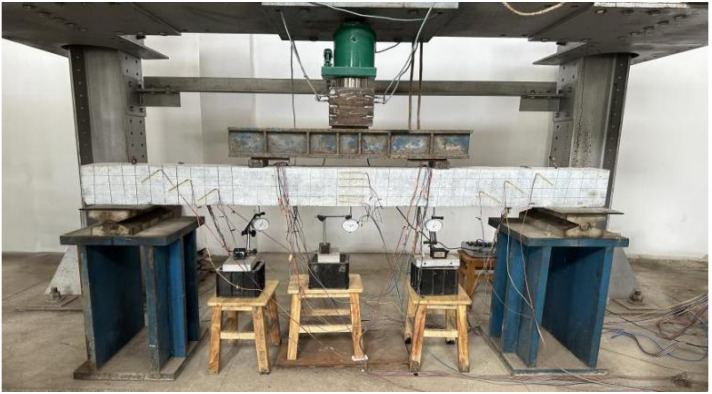
Schematic diagram of the test apparatus.

This test was a static load test. Manual hydraulic jacks were employed for stepwise loading, with an initial load increment of 10 kN per step. Upon the formation of visible cracks on the surface of the concrete beam, the load increment was reduced to 5 kN per step until the test beam failed. Each stage of loading and pressure stabilization lasted for 2 min. During this period, test data, including load, displacement, and strain, were recorded, and the crack morphology of the test beam was observed. The test setup is illustrated in Figure 5.

## 3. Test Results and Analysis

### 3.1. Analysis of Experimental Phenomena and Failure Modes

Static load tests were performed on a total of 21 reinforced concrete beams, grouped into seven categories. Based on the overall testing process, the force condition of the beams was categorized into four distinct stages. The first stage corresponds to the elastic phase, during which the stress–strain relationship of the beam remains linear. In this phase, both the concrete and reinforcing steel work together to bear the applied load, without significant deformation. The second stage is characterized by the initiation of concrete cracking. When the tensile stress exceeds the tensile strength of the concrete, cracks begin to form in the tension zone, and the reinforcing steel starts to assume the primary tensile force. The third stage represents the plastic phase, where the reinforcing steel yields, and the tensile capacity of the concrete progressively diminishes. This results in a substantial increase in beam deformation. Finally, the ultimate bearing stage is reached, during which the beam’s bearing capacity approaches its limit. At this point, the concrete in the compression zone fractures, leading to the eventual failure of the beam. Subsequently, a representative beam with distinctive force characteristics was selected from each test group for detailed analysis. The failure mode, crack propagation, and experimental phenomena of these beams are described and analyzed comprehensively.

**Figure 6:**
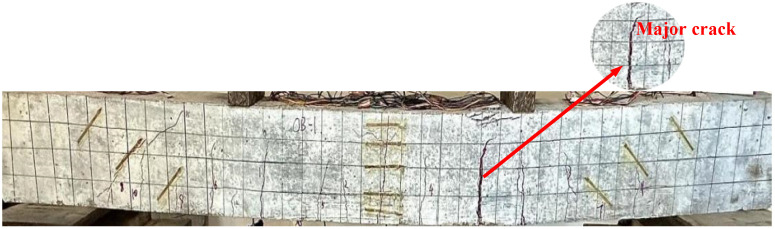
Failure of OB-1 beam.

The OB-1 beam was an unreinforced concrete beam. Prior to the commencement of the test, a preload of 10 kN was applied to ensure the proper functioning of the equipment and components. Formal loading was conducted using a stepwise incremental method, with increments of 5 kN at each stage. When the load reached 20 kN, the first fine vertical crack appeared at the left end of the mid-span of the beam, measuring approximately 62 mm in length and 0.1 mm in width. As the load continued to increase, both the number and width of the cracks expanded progressively. Notably, at a load of 54.5 kN, the width of the primary crack reached 0.5 mm, with its length extending to 104 mm. Upon reaching a load of 75 kN, the tensile reinforcement yielded, causing rapid crack propagation. At this point, the crack width increased to 2 mm, and the length extended to 157 mm, spreading toward the compression zone. Finally, under a load of 90 kN, the beam exhibited significant deflection, accompanied by the crushing of concrete in the compression zone, leading to structural failure. The maximum crack width recorded during the test was 5 mm. The failure pattern of the OB-1 beam is illustrated in Figure 6.

**Figure 7:**
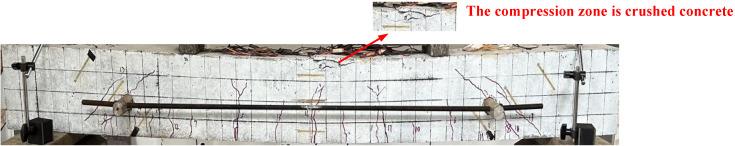
Failure of SRB-50-1 beam.

The SRB-50-1 beam was a side facade reinforcement beam subjected to an initial prestress of 50 kN. Upon initial loading, the beam remained in the elastic phase, without any visible cracks. When the load reached 35.5 kN, fine cracks first appeared on the right side of the mid-span, with a crack width of approximately 0.05 mm. Subsequently, as the load increased, both the number and width of the cracks gradually expanded. At a load of 60.6 kN, the first diagonal crack emerged at the support on the right end of the beam, measuring 0.1 mm in width and 120 mm in length. When the load reached 108.5 kN, the main crack widened to 0.5 mm and extended to a length of 142 mm. As the load continued to increase to 129 kN, the main crack expanded further to 5 mm, leading to the collapse of the concrete in the middle and upper portions of the span. The deflection significantly increased, and the beam ultimately failed at a load of 139.2 kN, reaching its ultimate bearing capacity. The failure pattern of the SRB-50-1 beam is illustrated in Figure 7.

**Figure 8:**
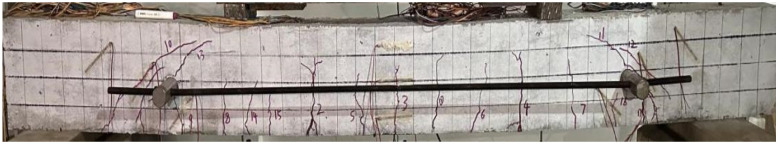
Failure analysis diagram of the SRB-75-1 beam.

The SRB-75-1 beam was a side facade reinforcement beam with an initial prestress of 75 kN. During the early stages of testing, no surface cracks or significant structural deformations were observed. When the applied load reached 39.8 kN, fine cracks first appeared in the right region of the mid-span, measuring 0.04 mm in width. Subsequently, these cracks propagated along the cross-section, with both their number and width gradually increasing. Upon the load reaching 59.7 kN, the first diagonal crack emerged at the left end support, with the main crack widening to 0.12 mm. As the loading continued to 118 kN, the main crack widened to 0.5 mm and extended to a length of 109 mm. At a load of 130 kN, the tensile reinforcement and prestressed tendons approached the yielding point, halting the increase in crack count but accelerating the expansion of crack width. Finally, at 134.8 kN, the main crack widened to 5 mm, accompanied by a significant increase in beam deflection and overall deformation. The failure occurred when the mid-span concrete crushed under a load of 141.4 kN, marking the ultimate load capacity of the beam. The failure pattern of the SRB-75-1 beam is illustrated in Figure 8.

**Figure 9:**
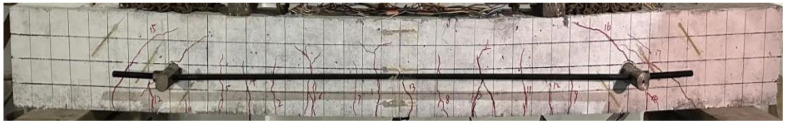
Failure analysis diagram of the SRB-100-1 beam.

The SRB-100-1 beam was a side facade reinforcement beam subjected to an initial prestress of 100 kN. During the initial loading phase, no significant cracks or deformations were observed on the surface of the beam. When the applied load reached 44.3 kN, a vertical main crack emerged at the mid-span with a width of 0.05 mm and a length of 26 mm. As the load increased, both the number and width of cracks progressively grew. At a load of 63.4 kN, the first inclined crack appeared below the drilling position at the left end of the beam, measuring 0.08 mm in width and 79 mm in length. Upon further loading to 72 kN, the tensile reinforcement began to yield. At 87 kN, the concrete strain gauge located at the lower part of the beam was damaged, and the reinforcing bars entered the strengthening stage. As the load reached 115 kN, the reinforcing bars transitioned into the necking stage, exhibiting pronounced plastic deformation. At 123.7 kN, the width of the main crack widened to 0.5 mm with a length of 98 mm, and the strain in the prestressed tendons intensified. Although the number of cracks stabilized, their widths continued to increase. Ultimately, when the load reached 137.5 kN, the width of the main crack expanded to 5 mm and its length increased to 192 mm. This was accompanied by the spalling of concrete fragments and a sharp increase in beam deflection. The concrete in the compression zone on the right side of the mid-span collapsed, marking an ultimate bearing capacity of 147 kN and resulting in structural failure. The failure pattern of the SRB-100-1 beam is illustrated in Figure 9.

**Figure 10:**
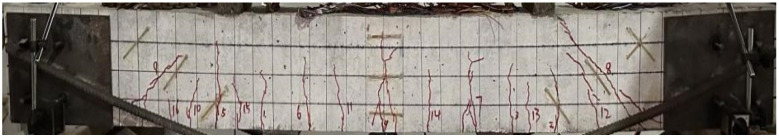
Failure diagram of the TRB-30-1 beam.

The TRB-30-1 beam was a conventionally reinforced concrete beam subjected to a prestress of 30 kN. During the initial loading phase, the surface of the beam structure remained intact, without any visible cracks or deformations. When the applied load reached 70.1 kN, the first vertical crack appeared at the left end support rod, measuring 0.04 mm in width and 33 mm in length. As the load increased, both the number and width of cracks gradually expanded. At a load of 83.5 kN, a primary crack emerged in the mid-span tensile zone, with a width of 0.06 mm and a length of 32 mm. Upon further loading to 107.4 kN, diagonal cracks developed at the right-end steel plate, characterized by a width of 0.11 mm and a length of 113 mm. At this point, the internal reinforcing bars entered the yield stage. The width of the primary crack increased to 0.24 mm and its length extended to 82 mm. When the load reached 143 kN, the concrete strain gauge sustained damage, and the reinforcing bars transitioned into the strengthening phase. At a load of 183.4 kN, the width of the primary crack widened to 0.5 mm with a length of 121 mm. Finally, at 208 kN, the crack propagated to a width of 5 mm and a length of 195 mm. The concrete in the compression zones at both ends of the mid-span was crushed, leading to the failure of the beam under a load of 230 kN. The failure pattern of the TRB-30-1 beam is illustrated in Figure 10.

**Figure 11:**
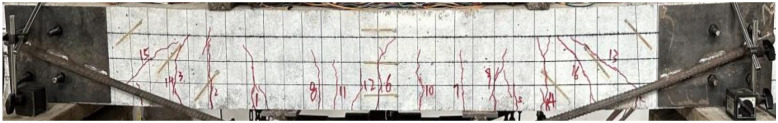
Failure diagram of the TRB-35-2 beam.

The TRB-35-2 beam was a conventionally reinforced concrete beam subjected to a prestress of 35 kN. During the initial loading phase, no visible cracks or deformations were observed in the beam. As the load increased to 72 kN, the first diagonal crack appeared near the left support rod, measuring 0.03 mm in width and 26 mm in length. With further load increments, at 84.3 kN, a primary crack developed at the mid-span, with a width of 0.05 mm and a length of 28 mm. Upon reaching a load of 109.8 kN, diagonal cracks emerged on the right steel plate, characterized by a width of 0.09 mm and a length of 107 mm. Concurrently, the main crack widened to 0.18 mm and extended to 57 mm in length. At a load of 154 kN, the strain gauge located at the bottom of the beam failed due to excessive deformation. When the load reached 186.8 kN, the main crack widened to 0.5 mm and extended to 102 mm in length. Ultimately, at a load of 210.3 kN, the main crack widened significantly to 5 mm and extended to 188 mm in length, accompanied by a rapid increase in beam deflection and substantial structural deformation. The concrete in the compression zone exhibited crushing, and the beam reached its ultimate bearing capacity and failed at a load of 231.2 kN. The failure pattern of the TRB-35-2 beam is illustrated in Figure 11.

**Figure 12:**
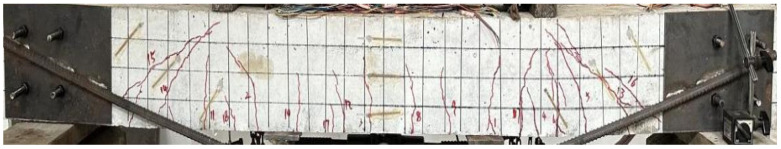
Failure diagram of the TRB-40-1 beam.

The TRB-40-1 beam was a conventionally reinforced concrete beam subjected to a prestress of 40 kN. During the initial loading phase, no significant deformation or cracking was observed in the beam. When the applied load reached 78.9 kN, an upward-extending crack first appeared at the location of the support bar on the right side of the beam, with a width of 0.04 mm and a length of 29 mm. As the load increased to 86.3 kN, a primary crack emerged in the mid-span tensile zone, characterized by a crack width of 0.05 mm and a length of 30 mm. Upon further loading to 113.5 kN, diagonal cracks developed on the right side of the beam, measuring 0.1 mm in width and 114 mm in length. Concurrently, the width of the primary crack increased to 0.15 mm, while its length extended to 46 mm. At a load of 138 kN, the concrete strain gauge located at the bottom of the beam failed. With the load reaching 191.8 kN, the width of the primary crack expanded to 0.5 mm, with a length of 87 mm. Ultimately, when the load increased to 214.7 kN, the width of the primary crack reached 5 mm, the length extended to 169 mm, and the deflection of the beam exhibited rapid growth, accompanied by substantial structural deformation. The beam ultimately failed when the ultimate bearing capacity of 232.8 kN was reached, resulting in the crushing of the concrete in the compression zone. The failure pattern of the TRB-40-1 beam is illustrated in Figure 12.

Overall, the unreinforced OB beams exhibited cracking at a load of 20 kN, whereas the SRB beams and TRB beams demonstrated delayed crack initiation, thereby enhancing the overall crack resistance of the structure. Moreover, even when cracks formed in the SRB beams and TRB beams under substantial loads, their widths were significantly narrower compared to those in the OB beams. Prestress effectively mitigated crack propagation by introducing a counteracting force. In comparison with the OB beams, the primary cracks in the prestressed reinforced beams after ultimate failure were finer, and the progression of cracking was more restrained. Additionally, as the level of applied prestress increased, so did the initial cracking load of the beams. Under identical loading conditions, the crack width exhibited an inverse relationship with the magnitude of the applied prestress and a direct correlation with the efficacy of inhibiting crack development. Furthermore, the overall deformation of the beam was reduced.

### 3.2. Flexural Capacity

This test defined the load value corresponding to the appearance of the first crack in the concrete beam as the cracking load, the load associated with plastic deformation or reaching the yield state of the beam as the yield load, and the load when the concrete beam reached its ultimate failure state as the ultimate load. As shown in Table 1, the cracking load, yield load, and ultimate load of the unreinforced OB beam were significantly lower than those of the SRB beam and the TRB-reinforced beam. By applying prestress on the side of the beam, the working performance of the SRB beams was enhanced compared to the OB beams. Depending on the magnitude of the initial prestress, the cracking load increased by 77% to 120%, the yield load by 55% to 85%, and the ultimate load by 53% to 62%. For TRB beams, the cracking load increased by 249% to 293%, the yield load by 175% to 214%, and the ultimate load by 152% to 155%. The effect of prestress was evident, as it not only improved the working performance of the beam but also enhanced its load-bearing capacity. However, with increasing prestress, while the cracking and yield loads of the SRB and TRB beams were significantly improved, the influence of prestress on the ultimate bearing capacity remained limited. Prestress alone cannot enhance the ultimate bearing capacity of reinforced beams. The increase in the ultimate bearing capacity of the SRB and TRB beams was attributed to the participation of prestressed steel bars in the load-bearing of the reinforced beam after reinforcement. The reinforcement effect of TRB beams surpasses that of SRB beams due to the greater distance between the force-applying axis of the prestressed tendons and the central axis of the concrete beam in TRB beams, resulting in a larger reverse bending moment. Additionally, the direction of prestress is altered through support rods at the bottom of the beam. In contrast, the prestressed long bolts in SRB beams are closer to the neutral axis of the beam, leading to a more limited reverse bending moment. Nevertheless, TRB consumes more materials and involves more complex construction processes compared to SRB.

### 3.3. Load-Reinforcement Strain Curve

**Figure 13:**
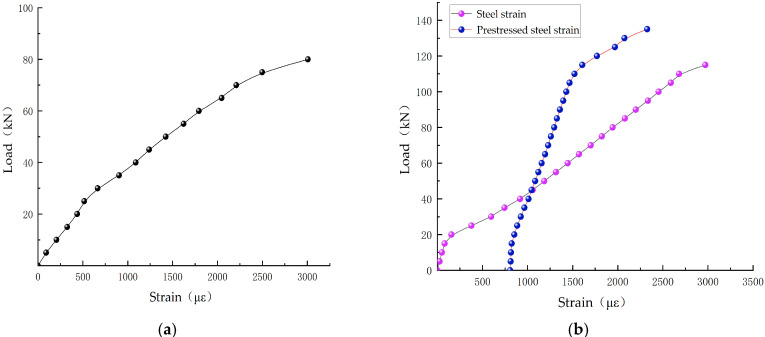

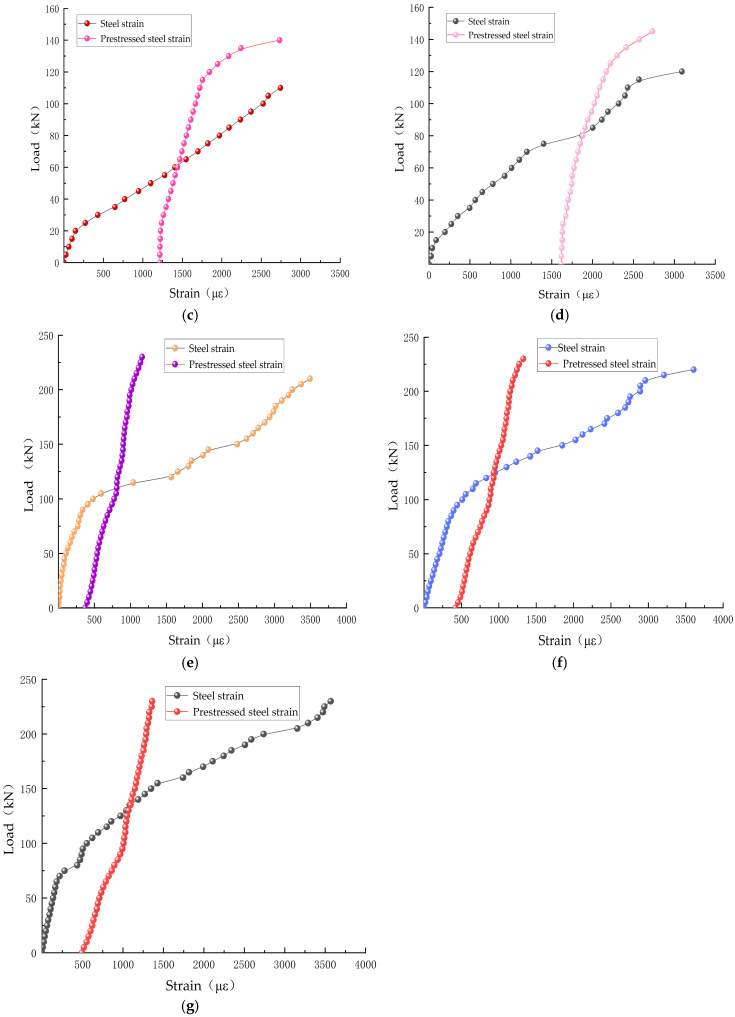
Comparative analysis of load–strain curves for reinforcing bars. (**a**) OB-1; (**b**) SRB-50-1; (**c**) SRB-75-1; (**d**) SRB-100-1; (**e**) TRB-30-1; (**f**) TRB-35-2; (**g**) TRB-40-1.

The load–strain curves of the reinforcing bars in concrete beams are presented in Figure 13. When the applied load was relatively low, the strain of the tensile reinforcement in the OB beams increased rapidly, transitioning into the yield stage and indicating a relatively low flexural stiffness. In the SRB beams, the implementation of prestress significantly enhanced the strain behavior of the tensile reinforcement, postponed the onset of yielding, and improved the overall flexural performance. Specifically, for the SRB-100-1 beam, the application of substantial prestress enabled the tensile reinforcement to remain in an elastic deformation state, even under significant loads, thereby effectively enhancing the stiffness and deformation control of the beam. As the level of prestress increased, the flexural stiffness of SRB beams progressively improved. In contrast, the prestressed tendons of the SRB-50-1 beams exhibited nonlinear deformation under lower loads, reflecting the limitations associated with insufficient prestress. Conversely, the prestressed tendons of the SRB-75-1 and SRB-100-1 beams maintained elastic deformation under higher loads, demonstrating superior stiffness and deformation control capabilities. The TRB beam, characterized by a uniform prestress distribution, exhibited a more gradual strain curve for both the tensioned reinforcement and the prestressed reinforcement. Prior to reaching the yield point, it could sustain a considerably larger load, further enhancing its overall bending resistance. Compared to the SRB beams, the prestressed tendons of the TRB beams continued to effectively counteract deformations induced by external forces under high loads, showcasing enhanced stiffness and anti-deformation properties.

## 4. Finite Element Analysis

### 4.1. Establishment of Finite Element Model in ABAQUS

#### 4.1.1. Finite Element Component Design

To achieve improved computational convergence, the concrete constitutive model employed the Concrete Damage Plasticity (CDP) model specified in Reference [29]. The CDP model integrates the principles of damage mechanics and plasticity theory, introducing damage variables to characterize the stiffness degradation of concrete. This approach effectively describes the cracking and crushing behavior of concrete under tension and compression, handles complex loading and unloading paths, simulates the nonlinear behavior of concrete, and is highly suitable for modeling real-world concrete structures. Additionally, constitutive models for the stress–strain relationships of the reinforcing bars and prestressed tendons were established in accordance with Reference [29].

The test beam models were categorized into three groups: one ordinary unreinforced OB beam, three traditional externally prestressed reinforced TRB beams with varying initial prestress levels, and three externally prestressed reinforced SRB beams located on the side facade. During the modeling process, C3D8 solid elements were employed along with solid shapes to accurately simulate the complex mechanical behavior of the concrete, ensuring high-precision results in the analysis. The steel reinforcement was modeled using T3D2 truss elements, which exhibit superior performance under significant tensile stress, while maintaining computational simplicity. Their wire-like geometry effectively captures the linear characteristics of the reinforcing bars and ensures compatibility with the surrounding concrete. Additionally, rigid spacers measuring 100 mm × 100 mm × 150 mm were placed at the beam’s support and loading points. These spacers were modeled using C3D8 elements with high rigidity to prevent deformation or damage, thereby avoiding simulation inaccuracies. To ensure the fidelity of the simulation, the model dimensions were kept consistent with those of the actual test beams. Following the completion of the model construction, material parameters were defined based on the properties of the various materials, and experimental data such as the mass density, elastic modulus, Poisson’s ratio, and plastic damage models were incorporated. After setting the material properties, the corresponding cross-sectional properties were assigned to each geometric component to ensure consistency with the test conditions.

#### 4.1.2. Initial Prestress Application

To apply the initial prestress value, a force is typically applied directly at the midpoint of the prestressed tendons. This operation is performed in the load module of ABAQUS. Specifically, the Initial Conditions option should be selected first, followed by choosing Predefined Field as the preloading method. Subsequently, under the “Type” category, the “Mechanical” type must be selected, along with the “Initial Strain” option from the “Mechanical Initial Conditions”. By entering the corresponding initial strain value, the prestress can be effectively applied to the prestressed tendons, thereby generating the desired initial prestress effect.

#### 4.1.3. Mesh Generation

**Figure 14:**
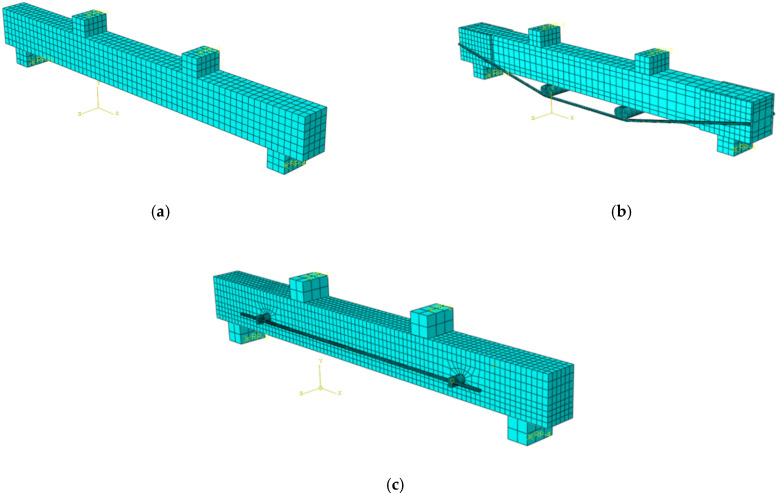
Mesh division diagrams for each model. (**a**) OB beam grid; (**b**) TRB beam grid; (**c**) SRB beam grid.

Common grid division methods encompass structured grids, swept grids, and free grids. Given the requirements of this analysis, the structured grid division method was selected. During the specific operations, the “Structured” division method was chosen within the “Mesh” module of ABAQUS, to ensure effective meshing of geometrically regular regions in the model. The grid size for the concrete beam was set to 30 mm, while the grid sizes for the steel bars and prestressed tendons were set to 10 mm. Additionally, the grid size for steel plates, support rods, and force transmission rods was also set to 30 mm. Since the pad blocks exerted a relatively minor influence on the overall structural force distribution, the specified grid size was deemed sufficient to meet the precision requirements. The grid division results are illustrated in Figure 14.

#### 4.1.4. Definition of Interface Contact and Boundary Conditions

The bond between steel bars and concrete was established through the “Embedded Region” method, which ensured precise stress transfer between the two materials. The interface between the spacer block and the concrete beam employed a coupled contact surface approach to guarantee accurate force transmission at the supports, thereby ensuring the reliability of the computational results.

In accordance with the testing requirements, boundary conditions for simply supported beams were defined. Within the Load module of ABAQUS, the boundary conditions were first set up, followed by the implementation of coupling constraints at the supports located at both ends of the simulated beam.

### 4.2. Post-Processing Analysis

**Figure 15:**
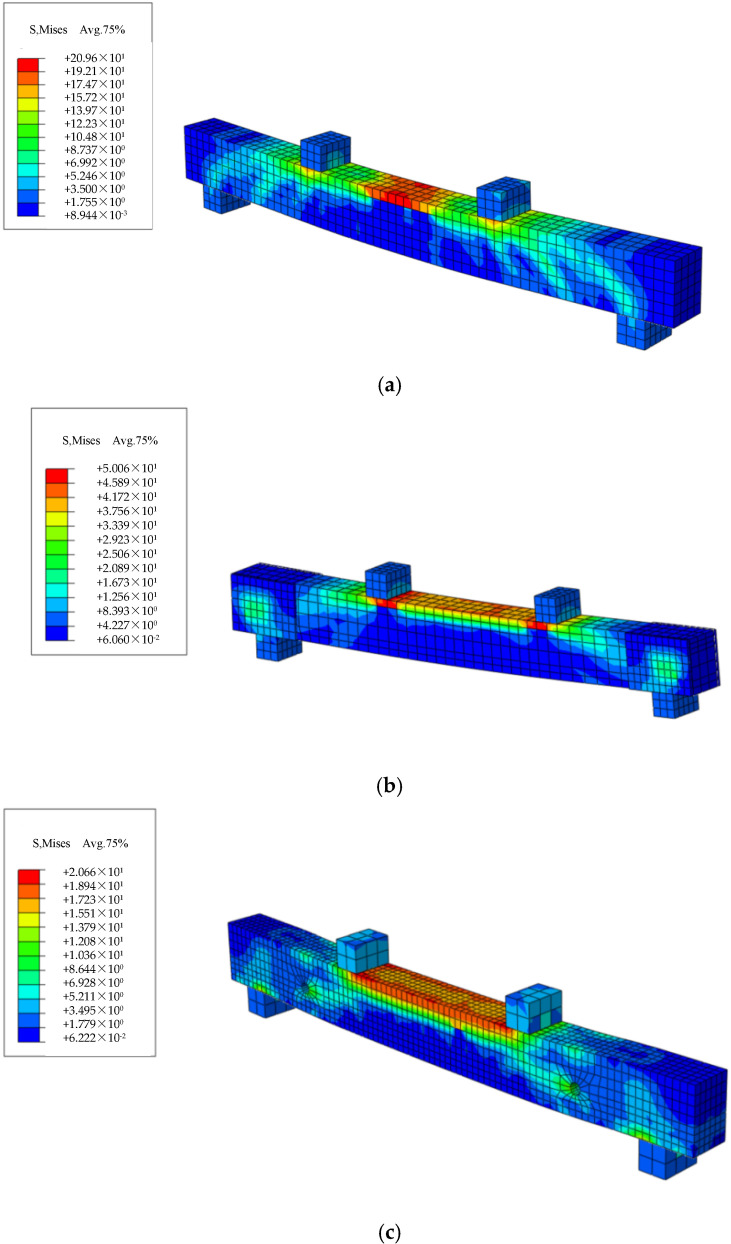
Cloud map of concrete stress in simulated beams. (**a**) 0B-1 beam; (**b**) TRB-35-2 beam; (**c**) SRB-75-1 beam.

**Figure 16:**
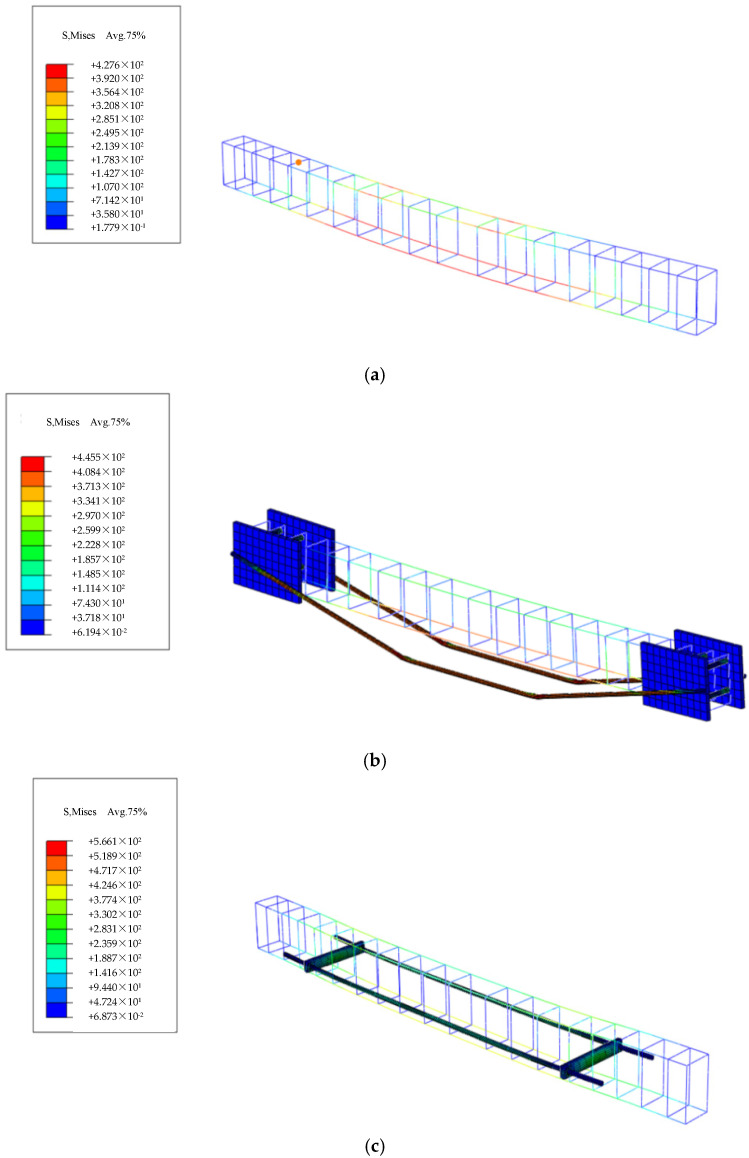
Cloud map of simulated beam reinforcement. (**a**) 0B-1 beam; (**b**) TRB-35-1 beam; (**c**) SRB-75-1 beam.

**Figure 17:**
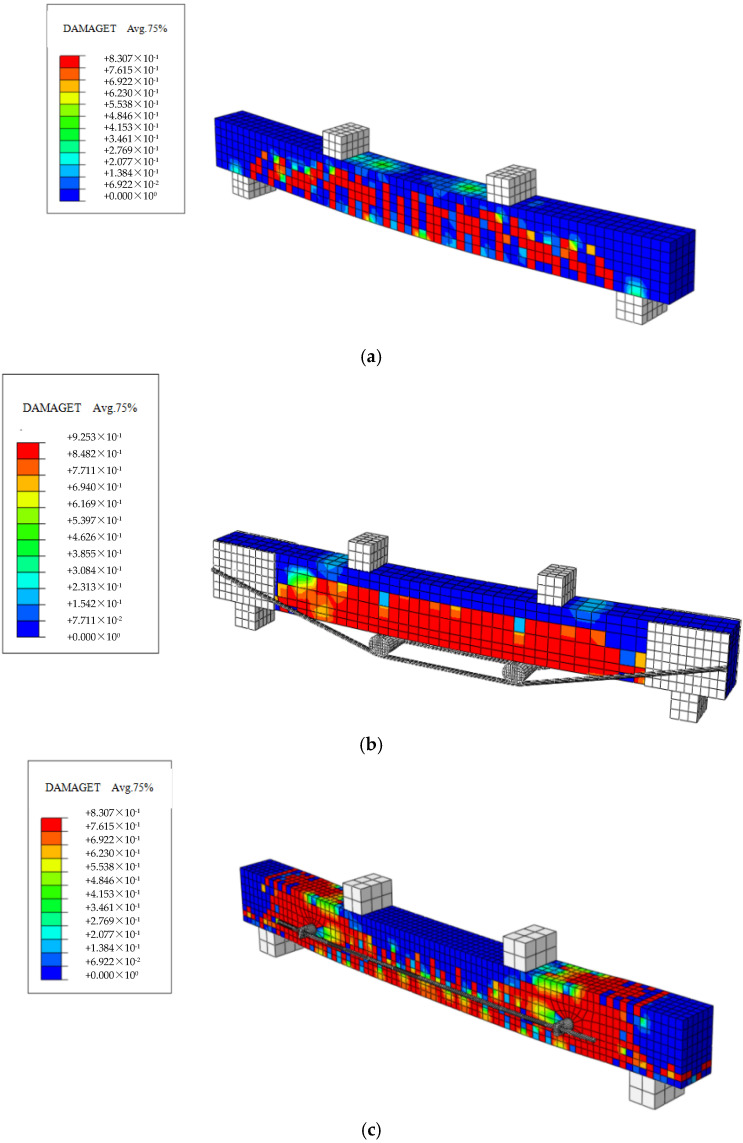
Cloud map illustrating the tensile damage stress distribution in simulated beams. (**a**) 0B-1 beam; (**b**) TRB-35-2 beam; (**c**) SRB-75-1 beam.

**Figure 18:**
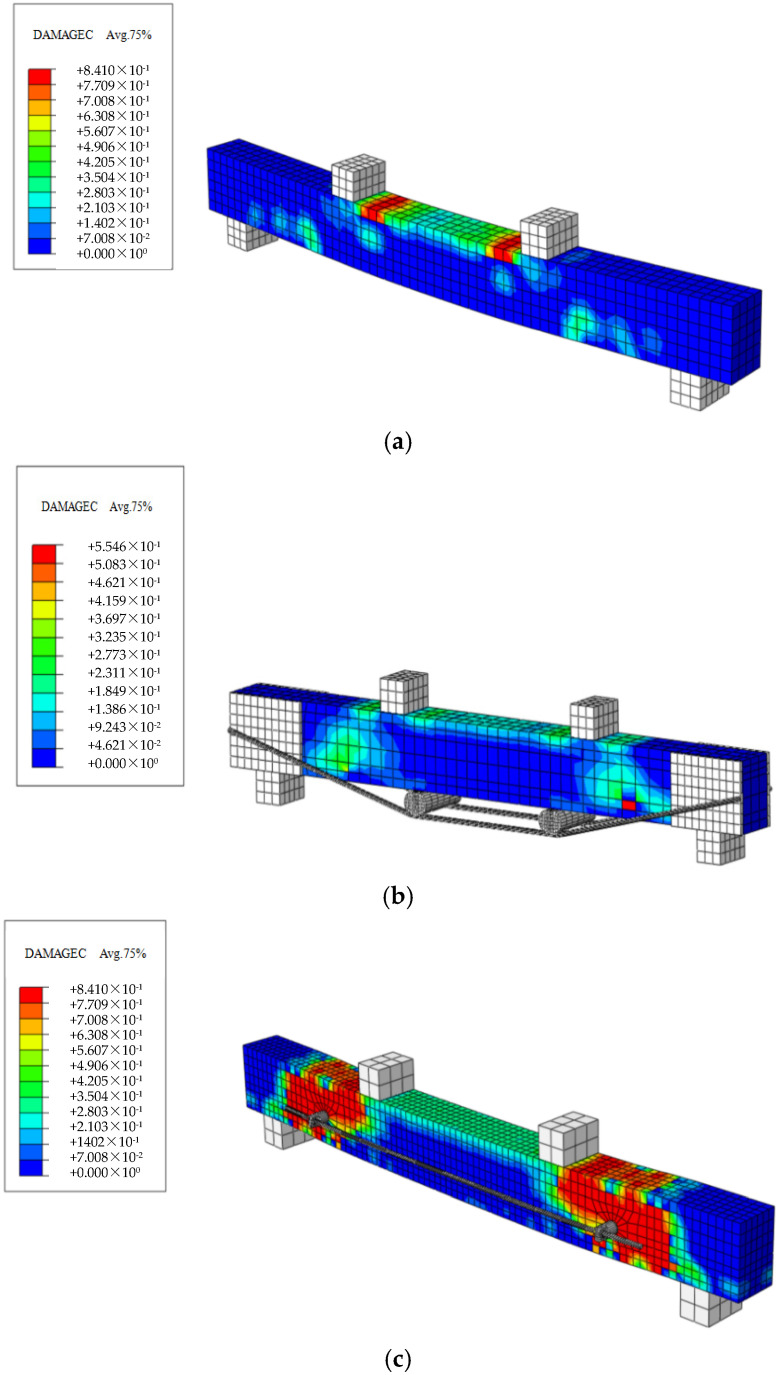
Stress cloud map illustrating simulated beam compressive damage. (**a**) OB-1; (**b**) TRB-35-2; (**c**) SRB-75-1 beam.

This paper presents the simulation of a total of seven beams, including ordinary reinforced concrete beams, traditionally prestressed reinforced beams with varying initial prestress values, and externally prestressed reinforced beams on the side facade. Tensile damage diagrams, compressive damage diagrams, concrete stress contour maps, and steel bar stress contour maps for these beams were individually simulated. The simulation results are presented in Figure 15, Figure 16, Figure 17 and Figure 18.

### 4.3. Comparison of Simulated Values and Experimental Values

To facilitate a clearer comparison between the test beam and the simulated beam, one representative beam was selected from each scheme. These beams were analyzed comparatively in terms of their load–deflection curves. Additionally, the ultimate bearing capacities of the test values and simulated values for all seven beams are presented in Table 2.

It can be observed from the table above that the finite element simulation results generally exhibited good agreement with the experimental data. The prediction of ultimate load was relatively accurate, with the error between the experimental and simulation values not exceeding 10%. After reaching the ultimate load, the overall experimental values for the SRB beam were higher than the simulated values, whereas the simulated values for the TRB beam exceeded the experimental results. This discrepancy can be attributed to the incomplete representation of material nonlinearity during the simulation process, particularly in the local failure phase. Once local failure or crack propagation occurred in the tested beams, the finite element model struggled to fully capture these intricate details.

## 5. Conclusions

This paper presents a study on the bending resistance testing of reinforced concrete beams with external prestressed reinforcement on the side facade, comparing these with ordinary unreinforced beams and traditionally prestressed reinforced beams. The research investigated multiple aspects, including the bending bearing capacity, concrete strain, strain in longitudinal and prestressed bars, mid-span deflection, failure mode, and crack development. Additionally, the ABAQUS finite element software was employed to simulate the behavior of the beams, providing a valuable reference for experimental prediction and theoretical calculation. Following a detailed theoretical analysis of prestress loss, a formula for flexural bearing capacity was derived. The preliminary primary conclusions drawn from this study are as follows:

(1) The external prestressed reinforcement technique can effectively enhance the flexural load-bearing capacity of concrete beams to a certain extent. Compared with the control beam, when initial prestress values of 50 kN, 75 kN, and 100 kN were applied, the ultimate load of the SRB beam increased by 53%, 55%, and 62%, respectively. Additionally, under the conditions of applying initial prestress values of 30 kN, 35 kN, and 40 kN, the ultimate load of the TRB beam increased by 152%, 153%, and 155%, respectively.
(2) The application of the external prestressed reinforcement method to the side facade significantly improved the workability of concrete. When initial prestress values of 50 kN, 75 kN, and 100 kN were applied, the cracking load of the SRB beam increased by 77%, 98%, and 120%, respectively, while the yield load of the SRB beam increased by 55%, 67%, and 85%, respectively. These results demonstrate the fundamental characteristic of prestress in enhancing structural performance.
(3) The reinforcement effect of traditional external prestressing is superior to that of side-facade external prestressing. The traditional external prestressed reinforcement method significantly enhances the working performance of concrete beams. When initial prestress values of 30 kN, 35 kN, and 40 kN were applied, the cracking load of the TRB beam increased by 249%, 265%, and 293%, respectively, while the yield load of the TRB beam increased by 175%, 185%, and 214%, respectively, thereby demonstrating the substantial effectiveness of prestressing. The significant difference in bearing capacity between SRB beams and TRB beams can be attributed to the fact that the traditional external prestressing structural layout is more conducive to achieving the desired prestressing effect. However, the TRB prestressing method is complex to implement, and drilling holes at the ends of concrete beams may introduce safety risks and pose insurmountable challenges.
(4) The ABAQUS finite element software analysis conducted on the three types of concrete beams indicated that the accuracy of the finite element software analysis and calculation met the requirements. The results of modeling and analyzing the test beams showed that the load–deflection curves of the simulation and the test were approximately consistent in their trend, and the ultimate bearing capacity of the simulation was basically consistent with the test results.

External prestressing on the side facade, as a new reinforcement method, is worthy of promotion. This reinforcement technology is convenient for construction, has simple equipment, requires less human and material resources, has a short construction period, and has significant economic benefits. Moreover, the reinforced prestressed tendons and their accessories are located on the side facade of the concrete beam, without reducing the clear height under the concrete beam.

## Figures and Tables

**Table 1:** Flexural bearing capacity (kN) of test beams.

No.	Cracking Load	Increase Range	Yield Load	Increase Range	Ultimate Load	Increase Range
OB-1	20.1	—	74.3	—	91.3	—
SRB-50-1	35.5	77%	115.4	55%	139.2	53%
SRB-75-1	39.8	98%	124.2	67%	141.4	55%
SRB-100-1	44.3	120%	137.6	85%	147.0	62%
TRB-30-1	70.1	249%	204.1	175%	230.0	152%
TRB-35-2	73.4	265%	211.7	185%	231.2	153%
TRB-40-1	78.9	293%	214.5	214%	232.8	155%

**Table 2:** Comparative analysis of test values and simulation values.

Specimen Identifier	Test Value (kN)	Analog Value (kN)	Tested Values/Simulated Values
OB-1	91.3	90.2	1.01
SRB-50-1	139.2	127.2	1.09
SRB-75-1	141.4	132.4	1.06
SRB-100-1	147	138.1	1.06
TRB-30-1	230	232.9	0.98
TRB-35-2	231.2	236.6	0.97
TRB-40-1	232.8	240.2	0.96

## Data Availability

The original contributions presented in this study are included in the article. Further inquiries can be directed to the corresponding author.
